# Community acceptance of reactive focal mass drug administration and reactive focal vector control using indoor residual spraying, a mixed‐methods study in Zambezi region, Namibia

**DOI:** 10.1186/s12936-021-03679-1

**Published:** 2021-03-22

**Authors:** Kathryn W. Roberts, Cara Smith Gueye, Kimberly Baltzell, Henry Ntuku, Patrick McCreesh, Alysse Maglior, Brooke Whittemore, Petrina Uusiku, Davis Mumbengegwi, Immo Kleinschmidt, Roly Gosling, Michelle S. Hsiang

**Affiliations:** 1 Malaria Elimination Initiative, Global Health Group, University of California, (UCSF), 550 16th St, San Francisco, CA USA; 2 Global Programs for Research and Training, Malaria Elimination Initiative Namibia, Windhoek, Namibia; 3 Department of Family Health Care Nursing, School of Nursing, UCSF, San Francisco, USA; 4 Department of Pediatrics, University of Texas, Southwestern Medical Center, 5323 Harry Hines Blvd, TX Dallas, USA; 5 National Vectorborne Diseases Control Programme, Namibia Ministry of Health and Social Services, Windhoek, Namibia; 6 Multidisciplinary Research Centre, University of Namibia, Windhoek, Namibia; 7 Wits Research Institute for Malaria, Wits/SAMRC Collaborating Centre for Multi-Disciplinary Research on Malaria, School of Pathology, Faculty of Health Sciences, University of the Witwatersrand, Johannesburg, South Africa; 8 Department of Infectious Disease Epidemiology, London School of Hygiene and Tropical Medicine, London, UK; 9 Department of Pediatrics, UCSF, San Francisco, USA; 10 Southern Africa Development Community Malaria Elimination Eight Secretariat, Windhoek, Namibia

**Keywords:** Malaria, Plasmodium falciparum, Malaria elimination, Namibia, Mass drug administration, Indoor residual spraying, Reactive case detection, Community acceptability, Qualitative and Mixed Methods

## Abstract

**Background:**

In Namibia, as in many malaria elimination settings, reactive case detection (RACD), or malaria testing and treatment around index cases, is a standard intervention. Reactive focal mass drug administration (rfMDA), or treatment without testing, and reactive focal vector control (RAVC) in the form of indoor residual spraying, are alternative or adjunctive interventions, but there are limited data regarding their community acceptability.

**Methods:**

A parent trial aimed to compare the effectiveness of rfMDA *versus* RACD, RAVC *versus* no RAVC, and rfMDA + RAVC *versus* RACD only. To assess acceptability of these interventions, a mixed-methods study was conducted using key informant interviews (KIIs) and focus group discussions (FGDs) in three rounds (pre-trial and in years 1 and 2 of the trial), and an endline survey.

**Results:**

In total, 17 KIIs, 49 FGDs were conducted with 449 people over three annual rounds of qualitative data collection. Pre-trial, community members more accurately predicted the level of community acceptability than key stakeholders. Throughout the trial, key participant motivators included: malaria risk perception, access to free community-based healthcare and IRS, and community education by respectful study teams. RACD or rfMDA were offered to 1372 and 8948 individuals in years 1 and 2, respectively, and refusal rates were low (< 2%). RAVC was offered to few households (n = 72) in year 1. In year 2, RAVC was offered to more households (n = 944) and refusals were < 1%. In the endline survey, 94.3% of 2147 respondents said they would participate in the same intervention again.

**Conclusions:**

Communities found both reactive focal interventions and their combination highly acceptable. Engaging communities and centering and incorporating their perspectives and experiences during design, implementation, and evaluation of this community-based intervention was critical for optimizing study engagement.

## Background

Malaria control efforts have led to declines in malaria transmission worldwide [1].

In Namibia, confirmed malaria cases decreased 97%, from over half a million in 2001 to 14,406 in 2011, corresponding to a decline in annual incidence from 422 to 11 cases per 1000 population [2]. To further target asymptomatic infections, in 2012, the National Vector-borne Diseases Control Programme (NVDCP) began implementing reactive case detection (RACD), where teams visit the homes of passively-identified malaria index cases and conduct testing and treatment among household members and neighbours [3–6]. However, reliance on conventional rapid diagnostic tests (RDTs) limits RACD’s effectiveness. In Namibia and other low endemic settings in southern Africa, RACD using RDTs only identifies up to one-third of infections detected by more sensitive molecular methods [4, 7]. The use of molecular tests to inform community care is impractical due to long turn-around times and cost [8].

An alternative to RACD is reactive focal mass drug administration (rfMDA), or presumptively treating people in active malaria foci. Reactive vector control (RAVC), in the form of indoor residual spraying (IRS), targets the mosquito and reduces human-mosquito contact. The effectiveness of rfMDA *versus* RACD, RAVC *versus* no RAVC, and rfMDA + RAVC *versus* RACD only, were compared in a two-by-two factorial design cluster randomized controlled trial [9] In adjusted analyses, rfMDA and RAVC implemented over a one-year period with over 80% coverage decreased malaria incidence by 48 and 52%, respectively, with their combination decreasing incidence by 74%.

Community acceptability is critical to intervention uptake, coverage, and sustainability. To understand acceptability of RACD, rfMDA, and RAVC, the study examined key factors of acceptability including: community engagement, attitudes, and beliefs and knowledge about malaria and the interventions [10–17].

## Methods

### Study site

The study was conducted in the western Zambezi region, Namibia, within 11 contiguous health facility catchment areas with an enumerated population of 33,418. Malaria transmission is seasonal and primarily due to *Plasmodium falciparum* [18]. From 2010 to 2015, annual case incidence was < 15/1000 population; incidence rose in 2016, reaching its peak at 40.2/1000 in 2017 [2, 19]. Community infection prevalence measured by highly sensitive loop-mediated isothermal amplification was 2.2% in 2015 [20]. Routine Namibia Ministry of Health and Social Services (MoHSS) interventions included case management, RACD, and annual pre-season IRS, using dichlorodiphenyltrichloroethane (DDT) on traditional structures and deltamethrin on modern structures, which represent a minority [21].

### Trial context

The trial aimed to evaluate the effectiveness of reactive focal interventions targeting the human and/or mosquito reservoirs of infection using rfMDA, RAVC, and their combination (rfMDA + RAVC). Trial design and results have been reported elsewhere [18]. Briefly, 56 enumeration areas (EAs) were randomized to receive rfMDA or RACD, with or without RAVC (Table 1). The 2 × 2 factorial design enabled assessment of individual interventions and their combination.

**Table 1:** 2 × 2 factorial design of parent trial

		Human intervention	
		RACD* (reactive case detection) *28 clusters*	rfMDA^†^ (reactive focal mass drug administration) *28 clusters*
*Mosquito intervention*			
No RAVC		**A**	**B**
(no reactive focal vector control)		RACD only arm	rfMDA only arm
*28 clusters*		*14 clusters*	*14 clusters*
RAVC^‡^		**C**	**D**
(reactive focal vector control)		RACD + RAVC arm	rfMDA + RAVC arm
*28 clusters*		*14 clusters*	*14 clusters*

*RACD (reactive case detection): administering rapid diagnostic tests to people living within a 500 m radius of an index case; treating positives with artemether-lumefantrine and single dose primaquine

^†^rfMDA (reactive focal mass drug administration): presumptively treating individuals living within a 500 m radius around an index case using artemether-lumefantrine, without testing

^‡^RAVC (reactive focal vector control): spraying long-acting insecticide, pirimiphos-methyl, on interior walls of sleeping structures in a 7-household radius around an index case

Reactive focal interventions were triggered when a malaria case was passively detected and confirmed by RDT (CareStart, AccessBio, USA) or microscopy at a participating health facility. If the index case resided in a RACD cluster, the team performed RDT-testing in the index case household and neighbours; if positive, they provided treatment with artemether-lumefantrine (AL, Coartem®, Novartis Pharmaceuticals, or Komefan 140®, Mylan Laboratories Limited) and single dose primaquine (Primaquine®, Remedica), per national policy. For index cases in rfMDA clusters, the team offered AL to the index case household and neighbours withoutesting. Both interventions aimed to reach ≥ 25 people within 500 m of the index case household, prioritizing those living closest. In RAVC clusters, the case household and the six closest households were offered IRS using micro-encapsulated pirimiphos-methyl (Actellic® 300 CS; Syngenta).

The acceptability study occurred during 2015 (pre-trial), 2016, and 2017 (years 1 and 2 of the trial). While data from 2016 were not included in the main trial analysis [9], the qualitative data from both years are included here, as interventions did not change year to year.

## Study design

This mixed-methods study included key informant interviews (KIIs), focus group discussions (FGDs), and an endline cross-sectional survey, nested within the above described cluster-randomized controlled trial [9, 18]. The qualitative assessment followed the theoretical framework of acceptability of Sekhon et al. including: burden (reasons for dropout), ethical consequences (reported adverse events), experience (user satisfaction), affective attitude (attitude towards intervention), opportunity costs (influence on adherence and participation), and intention (willingness to participate) [22]. Acceptability was measured quantitatively through refusal rates and reasons for refusal during trial implementation. An endline cross-sectional survey measured community-level acceptance rates and willingness to participate in future interventions. Participants were eligible if they were > 15 years old, spoke Silozi (the local language) or another language spoken by study personnel, resided in the study area, and provided informed consent.

Purposive and referral sampling were used to recruit participants for pre-trial KIIs. Key informants (KIs) included community leaders, non-governmental organization members (NGOs), and government representatives. After individual interviews, KIs supported referral sampling by suggesting KIs. Participants in pre-trial FGDs were community members from six randomly chosen health facility catchment areas in the study area. Two to four FGDs were conducted in each health facility catchment area, stratified by gender and three age categories: youth (15–18 years), young adult (19–35 years), and adult (> 35 years), except for two mixed-gender FGDs with NGO and MoHSS staff.

For KIIs and FGDs during Year 1 and Year 2, all residents of areas where trial interventions occurred were eligible, including those absent during the intervention or who refused to participate. FGDs were segregated by age: in Year 1 the age categories were: youth (15–18 years), young adult (19–35 years), and adult (> 35 years), in Year 2 these categories were collapsed due to challenges finding young participants: youth (15–25) years and adult (> 25 years). Youth FGDs were not segregated by gender due to a low number of available young males. This change was suggested and vetted by local staff, who were primarily young Zambezians. KIIs were conducted with community leaders, all of whom were adult males.

For the quantitative acceptability assessment, refusal rates were measured during trial implementation and willingness to participate in future interventions was assessed in 2017 in an endline cross-sectional survey. The sampling and methods of this survey have been described previously [9]. The survey was powered to detect anticipated differences in trial outcomes. In each of the 56 study clusters, around 25 households were sampled, providing a sample size of 4440 individuals. Those who reported participation in a community level intervention were asked about willingness to participate in future interventions. Data were analysed by the intervention to which they were randomized in 2017, and then restricted to individuals that reported receipt of a study intervention in 2017.

### Data collection

Pre-trial, study staff used semi-structured interview guides to explore malaria knowledge, risk perception, presumptive treatment impressions, community participation, and adherence optimization. During Years 1 and 2 of the trial, study staff used semi-structured interview guides to explore malaria and trial perceptions and experiences, intervention acceptability compared to MoHSS-delivered RACD, and possible improvements. One staff member led the FGD or KII and another took notes and created an audio recording, both of which were used to create English transcripts. Quantitative data were collected during trial implementation and the endline survey and recorded in Open Data Kit (ODK version 1.23.3).

### Data management and analysis

Pre-trial and Year 1 transcript data were analysed using NVivo (Version 11, QSR International, Melbourne) or Microsoft Excel (2016, Microsoft, Redmond, Washington). Year 2 transcripts were analysed in Dedoose (Version 8.0.35, Los Angeles, CA, USA: Sociocultural Research Consultants, LLC). Quantitative data collected were analysed in Stata (StataCorp. 2017. Stata Statistical Software: Release 15. College Station, TX: StataCorp LLC).

Analysis of pre-trial and Year 1 KIIs and FGDs was conducted by two coders, one of whom was Namibian, to ensure findings incorporated local context and knowledge. Analysis used an inductive iterative approach, allowing data to guide code creation. Each coder independently developed codes, which were compared and discussed to yield a unified code set. For year 2 data, two coders from UCSF used a deductive approach; a codebook was developed based on pre-trial and Year 1 themes to enable comparison across years. An inductive approach was allowed when new themes emerged. Coders analysed data from each data-collection phase then compared results across phases.

Refusal rates for rfMDA were compared to RACD, with or without RAVC. Refusal rates of RAVC with and without rfMDA are reported, but refusal rates for RAVC could not be compared to no RAVC. Refusal rates were measured at the individual level for rfMDA and RACD, and at the household level for RAVC. Refusal rates across arms were compared using logistic regression, adjusted for clustering.

## Results

Enrollment for the pre-trial, year 1, and year 2 KIIs and FGDs totaled 17 KIIs, 47 FGDs, and 449 FGD participants (Table 2). In years 1 and 2, 34 FGDs were conducted: RACD (6), RACD and RAVC (14), rfMDA (5), rfMDA and RAVC (9) (Table 3).

**Table 2:** Numbers of Key Informant Interviews, Focus Group Discussions, and Focus Group Discussion participants over study period, stratified by informant type and age category

	Pre-trial	Year 1	Year 2	All years
	N	N	N	Total
Key informant interviews				
Total interviews held	6	7	4	17
Participants				
Government or NGO	6	–	–	6
Community leaders	–	7	4	11
Focus group discussions				
Total discussions held	15	16	18	47
Participants	146	127	176	449
Youth	35	24	66	125
Adults	111	103	110	324

*NGO* non-governmental organizations

**Table 3:** Numbers of key informant interviews (KII), focus group discussions (FGD), and FGD participants by study arm

			Human Intervention					
			RACD (N)			rfMDA (N)		
			Y1	Y2	Total	Y1	Y2	Total
Mosquito Intervention								
No RAVC (N)								
KII			1	1	2	2	2	4
FGD			3	3	6	2	3	5
FGD participants			27	34	61	20	28	48
RAVC (N)								
KII			1	0	1	2	1	3
FGD			7	7	14	4	5	9
FGD participants			57	73	130	23	41	64

*Pre-trial excluded—study arms not assigned

### Pre‐trial findings

#### Healthcare-seeking behaviour

When asked where community members would go if they suspected malaria, most respondents mentioned public and private health facilities. Many respondents mentioned that elders may visit traditional healers. An MoHSS employee explained that a community member’s first response to malaria symptoms depends on healthcare access:

You look at the community that cannot afford to visit the hospital or the clinic because of the distance, they will probably start with the traditional healers, and from there that’s when they come to the clinics.

Despite the use of traditional medicine, a male FGD respondent said that “*recently it is evident that the difference between traditional healers and hospital is known… traditional healers cannot cure malaria disease*…” This distinction was seen consistently throughout the FGDs.

#### Possible barriers to participation

FGD participants believed that stigma could reduce participation. Some feared others would perceive them or their family as “unclean if a family member had malaria”. As one adult woman described,

But the… household who were found to be having malaria should also agree if they want other people to know… the community will label such a household as being untidy and not listening to what is being taught…

Comments about uncleanliness were made based on the environmental management educational program, which emphasized yard and household tidiness to prevent malaria.

Second, multiple participants mentioned human immunodeficiency virus (HIV) stigma and a perceived association between HIV testing and malaria testing. According to a female FGD participant,

Some would not go for [HIV] testing because they are afraid they might be found to have HIV; our people prefers to stay without knowing [un]til they see that they are very sick...

Participants explained that HIV tests have a similar appearance to malaria RDTs and community members could confuse the two and refuse.

Many respondents anticipated not wanting medication if they did not feel sick or had not tested positive for malaria. One male FGD participant stated,

It is not all right – medication is supposed to be for those who have been tested.

However, participants offered factors that could make presumptive treatment acceptable, such as sensitization. One man explained,

*People nowadays prefer to be tested by a doctor or nurse before accepting malaria dosage, though others may agree on condition that they are properly informed.*

All but one key informant said they would encourage others to accept presumptive treatment. However, three out of five health personnel key informants said they would refuse presumptive treatment, despite seeing the value for others.

Respondents anticipated reluctance about completing the full course of medication, explaining that some people might stop once symptoms diminished, or if symptoms did not subside immediately. Respondents mentioned that people may save medication to treat future illness, given limited access and distance from health facilities. To improve adherence, KII and FGD respondents suggested education, supervision, and reminders, especially for the sick, elderly, and those who cannot read.

Possible barriers to IRS were explored based on previous experience with the MoHSS. One woman discussed why people might decline, saying “*Others close their doors to those who spray against mosquitoes, claiming they bring cockroaches*”. However, several participants advised the inclusion of IRS, as one adult shared, “*Workers go to communities to spray against malaria and distribut[e] mosquito nets [which] needs to continue as the people really appreciate [it]”.*

#### Community sensitization

KIs and FGD participants suggested ways to engage the community for trial sensitization and ongoing engagement. According to an older male FGD participant,

…when an employed person tells you, people then listen. Those who went to school and are working tend to be influential. What they say is taken seriously.

Health extension workers, teachers, and nurses were identified to support community sensitization. Participants agreed that better understanding of interventions would likely lead to higher acceptance. Participants emphasized the necessity of involving the tribal council, or *khuta*, for community entry and sensitization.

### Year 1

#### Motivation to participate

Participants participated to receive protection from malaria via testing, treatment, and/or IRS; malaria prevalence made them fear infection. One woman explained, “*After seeing how much elders and kids were complaining of sickness was a reason enough for me to partake*.” That interventions were free, and that community engagement and sensitization efforts took place before and during the intervention, were described as positively influencing participation.

#### Perceptions of and attitudes towards reactive focal interventions

Intervention reception was overwhelmingly positive. Community-based care, the study team’s professionalism, and the respect shown for participants and local traditions were reported as critical for successful implementation. One man explained,

Most people are talking here that they have never seen people dedicated to their work like you showed us, you did not mind if the people were dirty or clean. You have treated them all equal… it’s the elders and the community leaders that are praising the most.

Suggestions for improvement included providing additional notice before community visits, arriving earlier, and processing participants and houses more quickly.

Criticisms included the lack of IRS in communities not receiving RAVC, lack of long-lasting insecticidal nets (LLINs), the need to remove furniture from households receiving RAVC, and a desire to receive additional medical interventions like tuberculosis and HIV testing and treatment. FGD participants reported that a neighbouring community felt jealous that they did not receive the same interventions, because they had been assigned to a different study arm. Such feelings can spread in communities and timely awareness provided the opportunity to address them.

#### Influences on participation and adherence

When asked why they participated, participants often shared that they or a relative was sick, or they knew people who had contracted malaria recently.

“I was told some people were coming to visit me the following day and I should prepare my house to get sprayed because all four of my grandkids had malaria.”

Here, family illness and advance notice positively influenced RAVC participation. Rationale for participation in RACD and rfMDA were similar; participants named recent illness and malaria education as motivators.

When FGD participants were asked their experience with malaria medication, most described positive experiences, such as this FGD participant:

There was no problem with the time of taking the prescribed dosage by the nurse, people drank or finished the medicine very well.

FGD participants reported that negative medication experiences did not affect participation or willingness to finish the medication.A few participants in three of the 16 FGDs raised concerns about treatment without testing. One FGD participant explained,

Some people were concerned, why are they giving us treatment? Do they think we are sick [with] malaria?

This concern was rare; most FGD participants were not concerned about treatment without testing and no one said they refused an intervention because they disliked the strategy.

#### Continued willingness to participate

When FGDs participants were asked whether they would be willing to participate in the same intervention in the future, two of 127 participants said they would not, referring to medical interventions. One rfMDA participant said, “*At least we should be tested first and only give medications to those who are found positive.*” The other, an RACD recipient, said he would decline because he did not need further intervention, “*I was already tested and I was negative, I don’t feel any sign of malaria*.”

The overwhelming majority of participants found the interventions acceptable. Reasons for future participation included: to protect their families, to know if they are sick, and medication effectiveness. Regarding RAVC, participants specifically referenced IRS effectiveness. One participant explained, *“… the spraying that you did, that was very nice. After you sprayed both flies and mosquito were no longer a lot and one could sleep even without a mosquito net*.” This visible change in mosquito presence was considered proof that RAVC was effective.

### Year 2

#### Motivation to participate

In Year 2, perceived malaria risk and the convenience of free community-based care and IRS continued to heavily influence participation. In one FGD, a woman commented, “*The disease is affecting us so much that is why we have decided to participate*,” which is noteworthy given the malaria outbreak the year prior (in 2016). Furthermore, one FGD participant remarked on the accessibility of the services,

“When you go to the clinic you will pay… you will find queues at the clinic, but the malaria team would come in the village to test and treat without you paying anything.”.

All four community leaders interviewed (KIs) confirmed that people appreciated no-cost, community-based interventions. FGD participants appreciated that the study teams visited twice to increase participation opportunities. Education by trial teams was important, as one participant explained, “…*always the nurse would explain the dose and the signs of malaria to the community; for the past six years we thought malaria is a headache, so now we know the difference*”.

#### Perceptions of and attitudes towards reactive focal interventions

Most participants responded positively to rfMDA, based on the belief that rfMDA protects people from illness.The majority of participants preferred rfMDA when asked whether rfMDA or RACD was preferable, based on their experience with one intervention and a description of the other. Concerns around RACD included: blood being used for satanic purposes or HIV testing, and that non-positive individuals miss the protective benefit of medication. Headmen were more neutral, stating that their primary concern was community health and were pleased with all interventions.

RAVC was generally perceived as a useful tool for malaria prevention. Participants in study arms that did and did not receive RAVC expressed a desire to have their houses sprayed against mosquitoes. Actellic CS was perceived to be “stronger” and more effective than DDT or Deltamethrin, the insecticides used by MOHSS. However, some participants noted strong smell, coughing, difficulty breathing, and itchy eyes with Actellic CS.

#### Influences on adherence and participation

Suggestions for improvement aligned with Year 1 findings, where residents and headmen asked that trial teams time visits better, communicate in advance, and provide mosquito repellants or LLINs. The majority of non-participants said they were unavailable during the intervention. However, some expressed a low malaria risk-perception, as one person said, *“[It was] my will not to participate because I am always exercising, so I will not get malaria*.” As in Year 1, a few community members described refusing because they disagreed with presumptive treatment. A community leader emphasized that refusal was likely due to lack of knowledge about malaria risk.

#### Continued willingness to participate

All but one FGD participant expressed willingness to participate in the same intervention in the future. All community leaders said they would welcome the intervention teams again. The lone dissenter, a male participant, had participated in rfMDA + RAVC and said, “*At least we should be tested first and only give medication to those who are found positive.*” Some participants preferred vector-based interventions over medical interventions. One woman explained, “*I don’t like tablets, maybe [I would participate] to have my house sprayed only, I don’t get healed when I drink tablets.*” A few other respondents agreed that combining medical and vector-based approaches, or delivering vector-based approaches only, were important to protect all community members.

### Refusal during trial implementation—years 1 and 2

RACD or rfMDA was offered to 1372 and 8994 individuals during years 1 and 2 of the trial, respectively. Refusal rates (Table 4) were low for both interventions in both years (< 2%), although higher for rfMDA than RACD. The refusal rate for RAVC was high at 13.9% in year 1. Refusals were due to lack of notification before arrival, and reluctance to move furniture at short notice. Few households (n = 72) were offered RAVC in year 1 due to staffing and transportation limitations (9). In year 2, when < 1% refused, more households were offered RAVC (n = 923) and advance notification was provided. RAVC could not be compared to no RAVC. RACD, offered at the individual level, could not be compared to rfMDA + RAVC, which was offered individual and household levels, respectively. All interventions met or exceeded the goal of 80% uptake among those offered participation.

**Table 4:** Refusal rates for RACD versus rfMDA among participants, and for RAVC among households

	Year 1			Year 2		
	N	Refused	p-value	N	Refused	p-value
RACD	894	3 (0.34%)	0.05	4711	10 (0.21%)	< 0.001
rfMDA	478	8 (1.7%)		4283	36 (0.84%)	
RAVC	72	10 (13.9%)	–	923	2 (0.22%)	-

### Willingness to participate in future interventions

2147 people participated in the acceptability portion of a cross-sectional survey in 2017, after the trial’s conclusion. 2024 respondents (94.3%) said they would participate in the same intervention again. Of RACD cluster respondents, 95.5% (1546/1619) said they would participate in a future round of RACD (Table 5). The most common reason for accepting RACD in the future was to know whether they were ill. The most common reason for refusing RACD in the future was that they recently tested negative for malaria. Of rfMDA cluster residents, 90.5% (478/528) confirmed future participation. Most commonly, they liked receiving free care and wanted to prevent and treat malaria. Those who would refuse rfMDA in the future were concerned about medication side effects. 98.7% (616/624) of RAVC cluster residents said they would participate again. The most common reason cited for future participation was to prevent mosquitoes and bugs.

**Table 5:** Primary reasons for willingness or unwillingness to participate in future interventions as assessed in an endline cross-sectional survey

	N	Willing	Unwilling	Don’t know/No response
RACD	1619	1546 (95.5%)	28 (1.7%)	45 (2.8%)
		Reasons:	Reasons:	
		714 (46.2%) To know whether they were ill	17 (60.7%) Tested negative for malaria recently	
		380 (24.6%) To know whether their children were ill	4 (14.3%) Afraid of needles	
		286 (18.5%) Care was free		
rfMDA	528	478 (90.5%)	31 (5.9%)	19 (3.6%)
		Reasons:	Reasons:	
		153 (32.0%) It is free	26 (83.9%) Worried about medication side effects	
		147 (30.8%) To both prevent and treat malaria	14 (45.2%) Do not want to take medication when not ill	
		105 (22.0%) Like to have their children treated		
RAVC	624	616 (98.7%)	7 (1.1%)	1 (0.16%)
		Reasons:	Reasons:	
		522 (84.7%) To keep mosquitoes and bugs away	2 (28.6%) No mosquitoes in area	
		89 (14.4%%) Structures were sprayed well during the previous visit		

Some participants provided multiple reasons

Of 528 participants who reported receipt of rfMDA, 77.5% (n = 409) rated rfMDA as equally or more acceptable than MoHSS RACD. Of 624 participants who reported receipt of RAVC, 97.4% (n = 608) found RAVC equally or more acceptable than MoHSS-delivered IRS.

## Discussion

rfMDA, RAVC, and their combination were at least as acceptable as RACD, the standard of care. Pre-trial, community members predicted the level of community acceptability more accurately than key stakeholders, who were doubtful that communities would accept study interventions, particularly rfMDA. Throughout the trial, participant motivators included: malaria risk perception, access to free community-based healthcare and IRS, and community education by respectful study teams. According to the theoretical framework of Sekhon et al. to evaluate acceptability of healthcare interventions, communities found the packages of interventions highly acceptable [22]. Refusal rates were low and participant attitude was largely positive. Community members understood the interventions and their purpose. While they had suggestions, there was no indication that they would refuse them in the future.

Ongoing community engagement and education was crucial to achieve and maintain acceptance. However, findings suggest the need for an even stronger approach, as exemplified in the interpretation of environmental management guidance as meaning that homes with malaria cases are “unclean” [23]. Providing malaria education during every visit ensured community understanding and the visits built trust between communities and the trial team. Nevertheless, some community members had misconceptions about malaria transmission and risk, which could be addressed with robust malaria education. Intervention teams can play a critical role in communicating messages and answering questions to address doubts; community engagement must be ongoing and iterative [23]. This work can be time and resource intensive, but soliciting community feedback through community meetings and ongoing community education should be implemented prior to the rollout of a large-scale, community-based trial or changes in the standard of care.

Pre-trial KII and FGD results informed pre-trial community sensitization, entry, and engagement strategies. For example, pre-trial FGDs and KIIs identified community leaders and frontline health workers as important information sources; the study team engaged them as “trial champions” to educate community members and facilitate community entry. The prediction by KIIs in the pre-trial phase that medication adherence could be a struggle informed the degree of time and emphasis placed on adherence. The qualitative data collection process served as an ongoing monitoring mechanism, both formally through FGDs and KIIs, and informally through discussions between community members and trial staff, enabling the team to address issues before they impacted enrollment or trust.

Compared to Year 1, the FGDs after Year 2 reflected a somewhat higher level of inquiry about the rationale and processes for treatment without testing. That rfMDA refusal rates were lower in Year 2 compared to Year 1 suggests that these questions were not connected to refusals, however, such comments could signal early intervention fatigue or that intervention familiarity enabled more questions.

Study limitations included the 2016 and 2017 malaria outbreaks, which likely increased malaria awareness and desire for services, possibly affecting intervention acceptance. Intervention coverage was low in 2016, particularly for RAVC, which limited the assessment of this intervention in Year 1. The lack of consistency in FGD age and gender segmentation limited group-based analysis. Finally, the perceptions of refusers were not captured, but they constituted a small proportion of the target population.

Strengths of the study included repeated acceptability data collection and immediate integration of results into trial procedures. Strategies to improve acceptability during this trial demonstrate that focusing on community acceptability can strengthen implementation and benefit targeted communities.

These findings are relevant for community-based implementation trials and interventions by Ministries of Health. High community acceptability and uptake are crucial for impact and sustainability. Integrating these findings into future programmatic and research approaches can facilitate community acceptability, particularly soliciting community feedback on past, planned, and ongoing interventions, focusing on engagement rather than just sensitization, providing ongoing education, and ensuring community needs are prioritized.

## Conclusions

Communities found both reactive focal interventions and their combination highly acceptable. Engaging communities and centering and incorporating their perspectives and experiences during design, implementation, and evaluation of this community-based intervention was critical for optimizing study engagement. Additional evidence of best practices for centering community perspectives and experiences in design, implementation, and evaluation is needed. Finally, future pre-intervention evaluations should be aware that community members may better predict community-level acceptability than key stakeholders.

## Data Availability

All data analysed for this article are co-owned by the Namibian Ministry of Health and Social Services and the University of Namibia. The de-identified data are available from the corresponding author upon reasonable request.

## Abbreviations

AL: artemether lumefantrine.
CRCT: cluster-randomized controlled trial
DDT: Dichlorodiphenyltrichloroethane
EA: Enumeration area
FGD: Focus group discussion
HIV: Human immunodeficiency virus
IRS: Indoor residual spraying
KI: Key informant
KII: Key informant interview
LLIN: Long-lasting insecticidal net
MoHSS: Ministry of Health and Social Services
NGO: Non-governmental organization
RACD: Reactive case detection
RAVC: Reactive focal indoor residual spraying
rfMDA: reactive focal mass drug administration
RDT: Rapid diagnostic test
